# Targeting PI3K/AKT/mTOR Signaling Pathway in Pancreatic Cancer: From Molecular to Clinical Aspects

**DOI:** 10.3390/ijms231710132

**Published:** 2022-09-04

**Authors:** Silviu Stanciu, Florentina Ionita-Radu, Constantin Stefani, Daniela Miricescu, Iulia-Ioana Stanescu-Spinu, Maria Greabu, Alexandra Ripszky Totan, Mariana Jinga

**Affiliations:** 1 Department of Internal Medicine, Dr. Carol Davila Central Military Emergency University Hospital, 051075 Bucharest, Romania; 2 Department of Gastroenterology, Dr. Carol Davila Central Military Emergency University Hospital, 051075 Bucharest, Romania; 3 Department of Family Medicine and Clinical Base, Dr. Carol Davila Central Military Emergency University Hospital, 051075 Bucharest, Romania; 4 Department of Biochemistry, Faculty of Dental Medicine, Carol Davila University of Medicine and Pharmacy, 050474 Bucharest, Romania

**Keywords:** pancreatic cancer, risk factors, tumor microenvironment, PI3K/AKT/mTOR, inhibitors

## Abstract

Although pancreatic cancer (PC) was considered in the past an orphan cancer type due to its low incidence, it may become in the future one of the leading causes of cancer death. Pancreatic ductal adenocarcinoma (PDAC) is the most frequent type of PC, being a highly aggressive malignancy and having a 5-year survival rate of less than 10%. Non-modifiable (family history, age, genetic susceptibility) and modifiable (smoking, alcohol, acute and chronic pancreatitis, diabetes mellitus, intestinal microbiota) risk factors are involved in PC pathogenesis. Chronic inflammation induced by various factors plays crucial roles in PC development from initiation to metastasis. In multiple malignant conditions such as PC, cytokines, chemokines, and growth factors activate the class I phosphoinositide 3-kinase (PI3K)/protein kinase B (AKT)/mammalian target of rapamycin (mTOR) (PI3K/AKT/mTOR) signaling pathway, which plays key roles in cell growth, survival, proliferation, metabolism, and motility. Currently, mTOR, AKT, and PI3K inhibitors are used in clinical studies. Moreover, PI3K/mTOR dual inhibitors are being tested in vitro and in vivo with promising results for PC patients. The main aim of this review is to present PC incidence, risk factors, tumor microenvironment development, and PI3K/AKT/mTOR dysregulation and inhibitors used in clinical, in vivo, and in vitro studies.

## 1. Introduction

Pancreatic cancer (PC) is the 14th most frequent form of malignancy and represents the seventh most common cause of cancer mortality in the world, having the highest incidence in Europe and North America [1,2]. Although in the past it was considered an “orphan” cancer type because of its low incidence [3], by 2050 it may become one of the leading causes of cancer death [4]. In 2012, 338,000 people were diagnosed with PC, making it the 11th most common cancer worldwide [5]. In the United States, PC represents the third leading cause of cancer-related death, following colon and lung cancer, with an increase in both incidence and mortality, and it is forecast to become the second most common cause of cancer mortality by 2030 [6]. In Australia, PC has the peak mortality among all main cancer types as the fourth most frequent cause of mortality [7].

The first pancreatic tumor was described in 1971 [8]. Pancreatic tumors can be divided into two groups: endocrine and exocrine tumors [9,10]. Multiple endocrine neoplasia type 1 (MEN1), von Hippel–Lindau disease (VHL), neurofibromatosis 1 (NF-1) (von Recklinghausen disease), and the tuberous sclerosis complex (TSC) are pancreatic endocrine tumors (PETs) [11]. Adenoma, cystadenoma, lipoma, fibroma, hemangioma, lymphangioma, and neuroma are benign non-endocrine pancreas tumors [9]. Moreover, scientific data revealed that the nervous system is involved in PC carcinogenesis [12], so pancreatic tumors can be neuroendocrine neoplasms [13]. Therefore, pancreatic neuroendocrine tumors or PNETs develop from the endocrine component of the pancreas [10]. Pancreatic ductal adenocarcinoma (PDAC) and acinar cell carcinoma are pancreatic exocrine neoplasms [10]. In addition, PDAC derives from pre-malignant precursor lesions, known as intraepithelial neoplasms (PanINs) [14], which is the standard nomenclature and diagnostic criteria for duct lesions classification [15]. Together with PanIN, intraductal papillary mucinous neoplasms (IPMNs) and mucinous cystic neoplasms (MCNs) are considered neoplastic precursors of the human PC [16].

PDAC is the most common type of PC, accounting for more than 90% of these neoplasms [17]. It is a highly aggressive malignancy, having a 5-year survival rate of less than 10% despite treatment [17]. This type of cancer is relatively uncommon, with an incidence of 8–12 per 100,000 per year and a lifetime risk of less than 3% of developing the disease; hence, the screening of asymptomatic adults is unfeasible [18]. Gastro-enteropancreatic neuroendocrine neoplasms (GEP-NENs) derive from neuroendocrine cells of the gastrointestinal tract [19]. Most of the PC cases arise from exocrine cells, while PC–endocrine cases including neuroendocrine tumors are uncommon [20]. 

The survival rate in PC remains low, with an improvement from <5% in 1990 to 9% in 2019 [18,21]. Currently, PC screening is not recommended for adult population because to its low incidence [22]. The only effective treatment of PC is the surgical resection of the tumor; therefore, an early diagnosis will improve the patient’s survival rate [23].

## 2. Pancreatic Cancer Risk Factors

Early diagnosis is of utmost importance in order to improve the prognosis of patients with PC, and it can be achieved by identifying the associated risk factors, taking into consideration that the presence of symptoms is not a screening criterion [24].

### 2.1. Non-Modifiable Pancreatic Cancer Risk Factors: Family History, Genetic Susceptibility, Age

An important PC risk factor is the family history [24,25], with an incidence of 7–10% [26,27,28,29]. First-degree relatives of patients with PDAC present a 1.5- to 3-fold higher chance of developing this pathology [30]. The presence of at least two first-degree relatives with PDAC defines familial PC, associated with genetic mutations including *ATM*, *BRCA ½*, *PALB2*, *CDKN2A*, *LKB1/STK11*, and *PRSS1* [24]. The risk ratio of developing PDAC is 6.79 times higher when a member of the family has the disease [24]. The presence of a hereditary disease such as hereditary non-polyposis colorectal cancer (Lynch syndrome), Peutz–Jeghers syndrome, familial adenomatous polyposis, or familial atypical multiple mole melanoma syndrome is another important risk factor for developing PDAC [24].

Moreover, the most common mutated genes in PC are *KRAS*, *CDKN2A* (encoding p16), *TP53*, and *SMAD4* [31]. In the case of patients diagnosed with PDAC, 95% of tumors have *KRAS* mutations [32]. These mutations accumulate early and are present in almost all PDAC cases [33], being associated with the progression of the disease [34]. PanIN lesions are characterized by *KRAS* codon 12, 13, or 61 mutations that induce multiple cancer effects [35]. In addition, *KRAS* mutations are considered a hallmark of the late-stage disease [36]. *CDKN24*, *TP53*, and *SMAD4* mutations are correlated with PanIN progression as follows, PanIN-1 (*KRAS*), PanIN-2 (*CDKN24*), and PanIN-3 (*TP53* and *SMAD4)*, and are usually observed in PDAC development [37]. The systematic review conducted by Tang B. and co-workers revealed that CDKN2A methylation is associated with a lower survival rate and is significantly increased in PC-PanIN patients [38].

Age represents another risk factor for PC, with most cases occurring in the adult population ranging between 70–80 years old [39]. Regarding sex, men are 30% more likely than women to develop PC [40].

### 2.2. Modifiable Pancreatic Cancer Risk Factors: Diabetes Mellitus, Diet, Smoking, Alcohol, Acute and Chronic Pancreatitis, Intestinal Microbiota 

There are also modifiable risk factors involved in PC development, including PDAC [34]. Diabetes mellitus is found in 25–50% of patients with PDAC years prior to the diagnosis, and the patients with new onset of diabetes mellitus having a greater risk of developing PC than long-term patients [34]. Some anti-diabetic medications may also represent a risk factor for PC [41]. However, this risk may be decreased by using oral hypoglycemic agents, in particular metformin. On the other hand, insulin can promote PC cell proliferation, thus augmenting the risk for neoplasia [34]. Therefore, diabetes mellitus is a predisposing factor for PC, augmenting the risk of the malignancy 1.5- to 2-fold [42]. The relationship between PC and diabetes mellitus can be considered unique [43]. It is believed that in almost 90% of PC cases, environmental factors, such as diet, play a crucial role [44]. The diet is responsible for about 50% of the cases [44]. Diets rich in animal proteins and fats and poor in antioxidants contribute to pancreatitis development and further to PC [45,46]. On the other hand, some studies revealed that red and processed meat consumption is not correlated with an increased risk of PC, while poultry intake may be a PC risk factor [47].

Smoking, obesity (body mass index > 30 kg/m^2^), and heavy alcohol use (>4 standard drinks/day) increase the risk of PC by less than five-fold [48]. Moreover, worldwide, 9% of all PC cases are related to smoking. Male smokers have an increased risk of 74% compared with non-smokers [49]. Tobacco contributes to 20–35% of PC cases. The exposure to carcinogens resulting from smoking such as nitrosamines and polycyclic aromatic hydrocarbons are responsible for mutations in proto-oncogenes *KRAS* and tumor suppressor *p53*. The risk of developing the neoplasm is proportional to the intensity and cumulative smoking dose and it remains higher than in a non-smoker even after 10 years of cessation [34]. The results of the meta-analysis conducted by Preziosi G. and his research team revealed that obese individuals have a higher risk (ranging between 1.19 and 1.47) of developing PC compared with people with normal weight [50]. Patients less than 50 years old may develop early onset pancreatic cancer, with an incidence of 10% of PC, with smoking and age of smoking initiation being important risk factors [51]. Heavy drinkers have an increased risk of 60% of developing PC versus non-drinkers, because of the formed acetaldehyde that acts as a carcinogen [52]. Nonetheless, if cigarette smoking has a negative impact, moderate alcohol consumption may have a protective effect [53].

The acute pancreatitis pathogenesis involves gallstones, medication, and genetic factors [54]. Moreover, the genetic aspect seems to be correlated with chronic pancreatitis [54], an important risk factor for PC [55], leading to endocrine and exocrine dysfunction [56]. Pancreatitis pathology is characterized by malabsorption, steatorrhea, fat-soluble vitamin deficiency, osteoporosis, and diabetes [56,57]. Patients diagnosed with chronic pancreatitis have a 14-fold risk of developing PC [58]. Hao L. and colleagues conducted a cohort study for a period of 13 years that included 1656 patients with chronic pancreatitis and investigated the link between chronic pancreatitis and PC development. Among all the patients included in the study, only 1.3% were diagnosed with PC, especially in the general population more than 60 years old and smoking history [59].

*Helicobacter pylori* infection and hepatitis B and C may also be risk factors for PC [29]. It is possible for these microorganisms to pass into the pancreas [60]. Moreover, oral periodontal pathogens (e.g., *Porphyromonas*, *Fusobacterium*, *Aggregatibacter*, *Prevotella*, or *Capnocytophaga*) can be involved in PC development [61]. Several epidemiological studies have reported a direct relationship between oral bacteria and PC [62]. Bo X. and his research team conducted a hospital-based case-control study that included 4821 participants (1392 were PC patients, and 3429 were controls) [63]. The study confirmed that age, diabetes, chronic pancreatitis, smoking, and family cancer history have been the primary risk factors for PC development [63]. Among all the risk factors mentioned above, age, diabetes, chronic pancreatitis, and smoking significantly increase the risk of PC development [63].

Zheng Z. and co-workers published in 2016 the results of a case-control study that included 646 volunteers, where 323 were PC patients and 323 were controls [64]. The results were similar to those found by Bo X. and confirmed that cigarette smoke, family history, obesity, and diabetes are risk factors for PC [64]. Furthermore, besides type 2 diabetes, non-alcoholic fatty liver disease (NAFLD) seems to be an independent PC-risk factor, while the use of aspirin and statins has a protective effect [65].

Zheng Z. and his research team evaluated the risk of PC on 650 patients submitted to chronic pancreatitis surgery [66]. The study reported the development of PC only in 12 patients (1.8%) after a median follow-up of 4.4 years [66]. Therefore, an early surgical intervention seems to have a protective role against PC development [66]. Moreover, chronic pancreatitis, PDAC, cystic fibrosis, and hemochromatosis are the main causes of type 3c diabetes [67].

Van Tran T. and his research team evaluated 196 Vietnamese patients (114 males and 82 females) diagnosed with PC regarding family cancer history, smoking, alcohol consumption, diabetes, inflammation disease, and HBV infection [68]. The study concluded that diabetes, smoking, and inflammation diseases are correlated with an elevated risk of PC [68].

Additionally, a relationship between psychological distress and PC has been observed [69]. Furthermore, chlorinated hydrocarbon compounds, pesticides, polycyclic aromatic hydrocarbons, metals, nitrosamines, radiation, and various airborne particles are risk factors for PC onset [70]. The results of a meta-analysis conducted by Seo M.S. reported that patients with systemic lupus erythematosus may have a risk of developing PC as well [71].

## 3. Tumor Microenvironment (TME) of Pancreatic Cancer

PC is a long, asymptomatic disease, so the development of initial carcinogenesis into invasive pancreatic carcinoma takes around 10 years. Therefore, in the absence of any screening method, this malignancy is discovered in late stages [72,73]. It has a high number of symptoms [74], with pain being frequent among PC patients due to pancreatic enzyme insufficiency, obstruction, and damage to the celiac plexus nerves [75]. Abdominal pain can have several causes, including tissue damage, inflammation, ductal obstruction, and infiltration [76]. Due to their abdominal localization, pancreatic tumors can be detected using computed tomography (CT), magnetic resonance imaging (MRI), or endoscopic ultrasound (EUS) even in early stages [72,77]. In the last decade, improvements have been achieved regarding PC chemotherapy, leading to a doubling in median overall survival rate [78].

The transformation of a normal cell into a malign one involves various mutations and epigenetic modifications [79]. The PC microenvironment contains fibroblasts, macrophages, and endothelial cells, increased numbers of extracellular matrix (ECM) compounds, elevated interstitial fluid pressure, and a high number of compressed tumor blood vessels [80]. Therefore, PDAC is characterized as a hard tumor [80]. In addition, pancreatic TME has an abundant fibrotic stroma, rich in various cell types and extracellular components including collagen, fibronectin, and hyaluronic acid [81]. Hyaluronan binds to its receptor CD44 and induces angiogenesis, epithelial-to-mesenchymal transition (EMT), and chemoresistance via receptor tyrosine kinase regulation and small GTPase [82].

Inflammation plays crucial roles in PC development from initiation to metastasis [83], leading to the activation of various inflammatory pathways [84]. Chronic inflammation induces the production of proinflammatory cytokines [85]. PDAC inflammation, which mediates TME development, involves exocrine acinar cells, ductal cells, and various stromal cells such as lymphocytes, neutrophils, mast cells, adipocytes, endothelial cells, and pancreatic stellate cells [86]. In a healthy state, stellate pancreatic cells are latent. In pancreatic pathological situations, stellate cells are capable of transitioning into a myofibroblast phenotype, playing a key role in the desmoplastic reaction [87]. PDAC stromal desmoplasia is the main histological hallmark, containing pancreatic stellate cells, ECM proteins, inflammatory cells, growth factors, and cytokines [88]. PDAC stromal desmoplastic reaction leads to the accumulation of a significant amount of type I collagen [89]. Moreover, via integrin α2β1, type I collagen is involved in PC cell adhesion, proliferation, and migration [82]. This cocktail of molecules and cells induces PDAC aggressiveness by promoting tumor growth, metastasis, and even chemoradiotherapy resistance [88].

**Figure 1:**
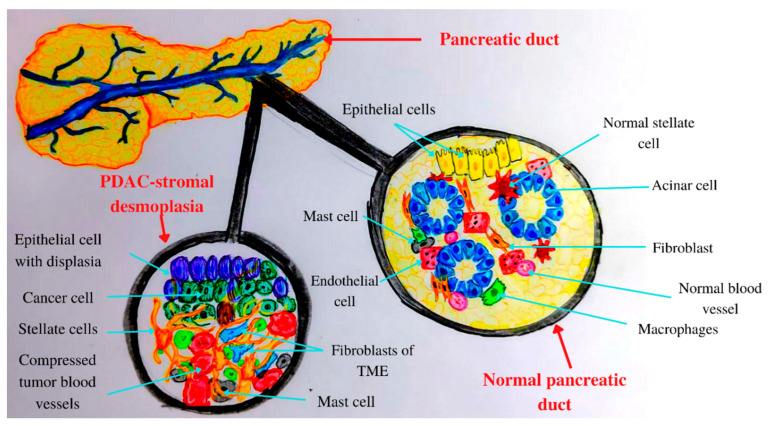
Anatomical and histological features of the pancreas: normal versus PADC.

In acute and chronic pancreatitis, acini, leukocytes, and stellate cells (Figure 1) contribute to pro-inflammatory cytokine release [90]. Therefore, PDAC chronic inflammation is caused by the risk factors mentioned above such as obesity, smoking, heavy drinking, low intake of fruits and vegetables, increased intake of fat, and a sedentary lifestyle [91]. The main mechanisms by which the microbiota influences PC development are chronic inflammation and antiapoptotic activity [92], regulation of the immune system-microbe-tumor axis, metabolism perturbation, and TME alteration [61].

Moreover, in the interstitial space of the tumor, PDAC patients have an excessive production of ECM proteoglycans and glycosaminoglycans [93]. ECM molecules that are secreted during tissue remodeling, angiogenesis, and tumor growth can modulate various cancer processes such as growth, metastasis, angiogenesis, differentiation, and immune response [94].

In PDAC, cancer-associated fibroblasts via various cytokines have an activated phenotype, promoting inflammation, angiogenesis, tumor growth and proliferation, invasion, metastasis, and regulation of the tumor metabolism [95]. The immune system is also involved, releasing cytokines, chemokines, and growth factors, which can mediate the neoplastic transformation [79]. Inflammation and insulin resistance, associated with obesity and type 2 diabetes, can enhance KRAS activation [96]. Moreover, a high fat diet induces *KRAS* activation, transforming a normal pancreatic cell into pancreatic intraepithelial neoplastic lesions [96]. Furthermore, TME orchestrates the tumor cell transcription programs [97]. EMT occurs in response to the activation of several pathways, such as receptor tyrosine pathways and TGF-β [98].

In the periphery of PDAC, tumor-associated macrophages are accumulated, being correlated with a M2 phenotype. This type of phenotype is characterized by extra-pancreatic and lymph vessel invasion, lymph node involvement, and shortened survival time [99]. The adipose tissue may also contribute to PC development via adipocytes, immune cells, endothelial cells, fibroblasts, stem cells, or progenitor cells [100]. Many of these cells can secrete pro-inflammatory cytokines (TNF-α, IL-6, leptin, TGF-β), which are known to be involved in cancer proliferation [100].

Exosomes, which are involved in the transport of proteins, lipids, and nucleic acids between cells, may be secreted by tumor cells including PC. PDAC tumor exosomes are involved in the establishment of the immunosuppressive microenvironment [101].

Among screening biomarkers, serum carbohydrate antigen 19-9 (CA 19-9) is the most used biomarker for PC, with a median sensitivity of 79% (70–90%) [102]. Kurahara H. and his research team detected CA 19-9 and p53 in 115 patients (80 with upfront surgery and 35 who received neoadjuvant therapy for resectable PC) [103]. These two biomarkers were statistically elevated in resectable PC patients with early recurrence after upfront surgery [103]. N^6^-methyladenosine (m^6^A) is the most recurrent RNA modification found in eukaryotes and is involved in the regulation of many cellular and biological processes and detected in various malignancies including PC [104].

## 4. PI3K/AKT/mTOR and Pancreatic Cancer

Cells and tissues communicate with each other by binding of various molecules—such as insulin, glucose, growth factors, cytokines [95], integrins, B and T cell receptors [105], hormones, and chemokines—to the tyrosine kinases (RTKs) or G-protein receptors (GPCRs) at the cell surface. This leads to the activation of intracellular signaling pathways, such as the class I phosphoinositide 3-kinase (PI3K)/Protein kinase B (AKT)/mammalian target of rapamycin (mTOR) signaling pathway [106,107,108].

PI3Ks are divided into three classes [109], each having four homologous regions, where the kinase domain is the most conserved [110]. In addition, PI3K class I has two subclasses, PI3K class IA that is activated by RTKs and PI3K class IB which is activated by GPCRs [111], and both are implicated in cancerous cell growth, survival, and invasion [112]. PI3K phosphorylates phosphatidylinositol 4,5-bisphosphate (PIP2) to phosphatidylinositol 3,4,5-trisphosphate (PIP3), leading to AKT activation [113]. PTEN is a lipid phosphatase that acts on PIP3, causing its dephosphorylation, thus being considered the negative regulator of the signaling pathway [114].

AKT activation consists of two phosphorylation processes, one performed by mammalian target of rapamycin complex 2 (mTORC2) at serine 473 residue, and the second one at threonine 308 residue performed by phosphoinositide-dependent kinase 1 (PDK1) [115]. In eukaryotes, AKT kinase has three isoforms, AKT1, AKT2, and AKT3, with high homology [115]. The first two isoforms are ubiquitously expressed. AKT2 is predominantly found in insulin-responsive cells, while AKT3 seems to be found mostly in neurons and testes [116]. *AKT1* and *AKT2* gene mutations are implicated in insulin resistance and further in diabetes pathogenesis [117]. AKT activation induces tuberous sclerosis protein 2 (TSC2) inhibition [118] and disrupt its interaction with TSC1 [119]. TSC1 is involved in cell growth and proliferation, survival, and autophagy [120]. Once activated, AKT induces protein synthesis and cell growth by mTOR activation and TSC1 and TSC2 inhibition [121].

mTOR has two complexes: mTORC1 complex, composed of mTOR, raptor, mLST8, and PRAS40, and the second complex mTORC2, which is formed by mTOR, rictor, mLST8, and Sin1 [122]. mTOR regulates translation by the phosphorylation of ribosomal protein S6 kinases (S6K) and 4E-binding protein 1 (4E-BP1). Activation of 4E-BP1 induces the release of the eukaryotic translation initiation factor 4E (eIF4E) [123]. The mTOR pathway is the main regulator of the mammalian metabolism that regulates cell growth and proliferation, protein synthesis, and autophagy [124]. mTOR plays an important role in apoptosis regulation, stimulating the cell growth [112] and immune cell regulation, differentiation, and functions [125]. Growth factors, nutrient energy, and even stress may activate or inactivate mTOR, which induces normal or dysregulated processes [126].

In the pathogenesis of PC, several key signaling pathways are involved, such as PI3K/AKT/mTOR [127] and Notch, Wnt, and Hedgehog [128]. Overexpression of the PI3K/AKT/mTOR pathway has been observed in a large number of malignancies including PC [129,130,131,132,133,134,135]. In addition, PI3K/AKT/mTOR is one of the most mutated signaling pathways in human cancers, usually correlated with the loss of PTEN and mutations in PIK3CA (encoding PI3K-p110α) and AKT1 [136]. PTEN expression is lost in 25–70% of all PC cases [137]. Furthermore, KRAS mutation induces overexpression of mutant PI3KCA [112].

One of the numerous signaling pathways activated by KRAS mutation is PI3K, which is also stimulated by various cytokines and growth factors through the RTKs [112]. The overexpression of AKT1, which stimulates cancer cell growth and proliferation, was found in 10–20% of the patients with PDAC [112]. During tumorigenesis, AKT1 may have opposite effects, may act as a pro-oncogenic factor suppressing apoptosis, or may restrict the tumor invasion [138]. Regarding AKT2, this isoform promotes cell migration and invasion, while AKT3 is involved in tumor migration [138].

**Figure 2:**
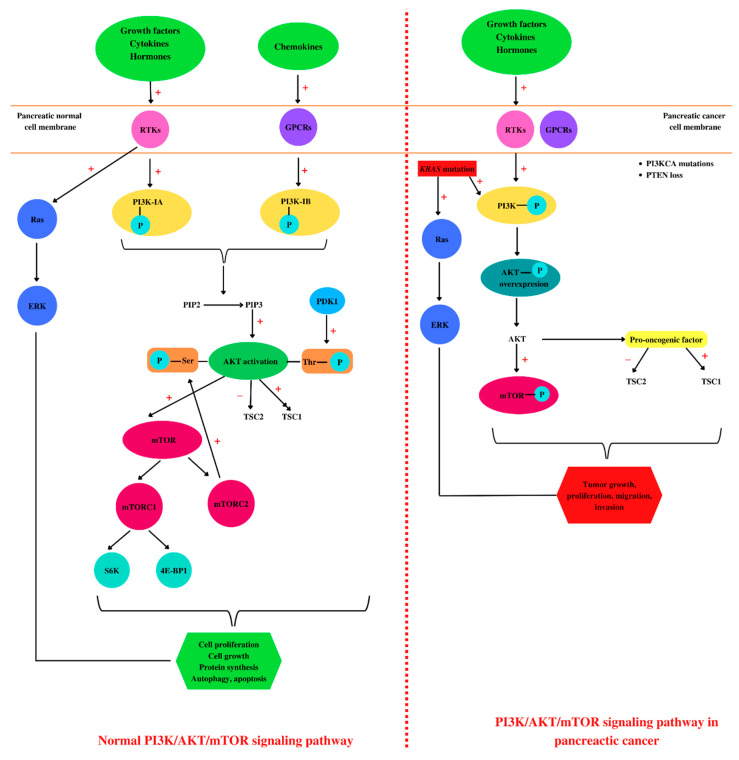
Pancreatic PI3K/AKT/mTOR: normal versus pathological. In a healthy cell, such as a pancreatic cell, different ligands (growth factors, hormones, cytokines, or chemokines) bind to tyrosine kinase (RTKs) or G-protein receptors (GPCRs) located in the cell membrane, leading to PI3K activation by phosphorylation. Further, PI3K catalyzes the phosphorylation of phosphatidylinositol 4,5-bisphosphate (PIP2) at phosphatidylinositol 3,4,5-trisphosphate (PIP3), leading to AKT activation. AKT activation consists of two phosphorylation processes, one performed by mammalian target of rapamycin complex 2 (mTORC2) at serine 473 residue and the second one at threonine 308 residue performed by phosphoinositide-dependent kinase 1 (PDK1). Phosphorylated AKT induces further activation of mTOR and tuberous sclerosis protein 1 (TSC1) and TSC2 inhibition. In addition, mTOR phosphorylates ribosomal protein S6 kinases (S6K) and 4E-binding protein 1 (4E-BP1). The binding of growth factors to RTKs leads to Ras activation and in the end to extracellular signal-regulated kinase (ERK) activation, correlated with cell proliferation. Altogether, ERK and AKT activation are conducive to cell growth, proliferation, protein synthesis, autophagy, and apoptosis. In pancreatic cancer cells, besides growth factors, hormones, and cytokines, KRAS mutations activate PI3K and Ras. Moreover, these types of cells are characterized by PIK3CA mutations and PTEN loss. All these molecular events are associated with an overexpression of AKT phosphorylation, regarded as a pro-oncogenic factor. Together with ERK, AKT induces tumor growth, proliferation, migration, and invasion. “+”: activates, “−”: inhibits.

Therefore, the PI3K/AKT/mTOR signaling pathway plays crucial roles in cell survival, growth, proliferation [139], metabolism, and motility (Figure 2) [140]. Moreover, these protein kinases, through phosphorylation processes, are involved in various cellular functions such as transcription and translation [141]. AKT is involved in cell survival and apoptosis by regulating some pro-survival and anti-apoptotic proteins Bcl-XL and NF-kB in both normal and cancerous cells, so targeting this molecule is considered a promising therapeutic approach for the treatment of pancreatic ductal adenocarcinoma [142].

**Figure 3:**
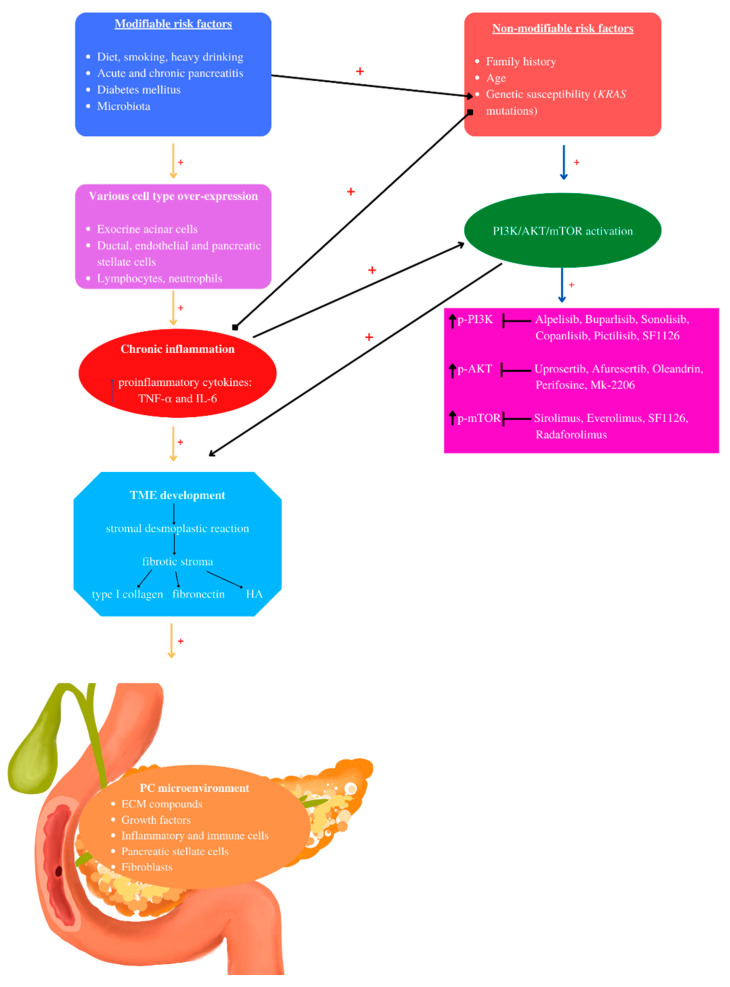
Pancreatic cancer pathogenesis: The pathogenesis of PC involves non- and modifiable risk factors that induce chronic inflammation mediated by several cell types. Chronic inflammation mediates TME development characterized by stromal desmoplastic reaction—the main PC histological hallmark, leading to fibrotic stoma formation, rich in type I collagen, fibronectin, and hyaluronic acid (HA). *KRAS* mutations may be activated by inflammation and modifiable risk factors such as diet and diabetes mellitus. Chronic inflammation and *KRAS* mutations are the main actors that induce PI3K/AKT/mTOR activation, contributing to PC-TME development, correlated with increased phosphorylated levels. Currently, the mentioned PI3K/AKT/mTOR inhibitors have been used in clinical studies. “+” activates.

In pathological conditions, cytokines, chemokines, and Fc receptors activate the PI3K/AKT/mTOR signaling pathway [143]. Vascular endothelial growth factor (VEGF), IGF-1, FGF, platelet-derived growth factor (PDGF), and TGF-α and -β bind to RTKs and activate PI3Ks [109,144,145]. VEGF and PDGF induce angiogenesis in neuroendocrine tumors, contributing to neuroendocrine tumor development [146]. PI3K, AKT, and mTOR are the three major nodes that suffer dysregulation and induce cancer progression [147]. Moreover, genomic aberrations activate PI3K in various PC lineages [148] (Figure 3).

Prorenin receptor (P)RR is a single transmembrane protein encoded by the *ATP6AP2* gene located on the X chromosome [149]. (P)RR is widely expressed in the brain, pancreas, heart, liver, placenta, and kidney, with this expression being significantly higher in various human cancers. (P)RR may activate PI3K/AKT/mTOR via angiotensin-II-reactive oxygen species formation [149]. The aldehyde dehydrogenase 1 family member A3 (ALDH1A3) is a crucial enzyme for glucose metabolism that can increase the expression of hexokinase 2 and further enhance glycolysis in PDAC cells. During pancreatic pathogenesis, glucose metabolism may promote PDAC metastasis both in vitro and in vivo [150]. Therefore, Nie S. et al. detected that ALDH1A3 is overexpressed in vitro and leads to increased activity of PI3K/AKT/mTOR [150].

In the liver, increased insulin levels enhance insulin-like growth factor-1 (IGF-1) synthesis and down-regulate IGF-1 binding proteins, leading to cell proliferation and an increased risk of PC [151]. Yan X. and his research team evaluated the expression of EG-VEGF in PC tissues and cells. The study reported high expression of EG-VEGF in PDAC, both cells and tissues. Moreover, the inhibition of EG-VEGF inhibits PC cell proliferation and promotes apoptosis via PI3K/AKT/mTOR [152].

Su CC measured the levels of EGFR, IGF1R, VEGFR, PI3K, AKT, mTOR, and PTEN in MiaPaCa2 human PC cells and observed their increased expression [153]. Lin S. and co-workers obtained similar results regarding PI3K, p-AKT, and p-mTOR using human PC cell lines (PANC-1). Moreover, the study reported that PC cell line supplementation with IGF-1 significantly increases the expression of the three proteins mentioned above [154]. Poor PC prognosis is linked to AKT phosphorylation at S473 [155]. Singh BN and co-workers detected increased phosphorylated levels of AKT and mTOR in human PC stem cells [156]. Liu X. and his research team measured elevated activity of PI3K/Akt/mTOR pathway in tissue samples of 54 PDAC patients [157]. In addition, serine/threonine kinase 33 (STK33) is involved in pancreatic neuroendocrine tumor (PNET) growth and progression via PI3K/AKT/mTOR pathway activation. Thus, STK33 was overexpressed in PNET specimens compared with normal pancreatic tissue [158].

## 5. PI3K/AKT/mTOR Inhibitors and Pancreatic Cancer

PI3K pathway mediates signals involved in cancer survival and progression and is also essential for other cancer processes such as endothelial cell angiogenesis, T cell differentiation, and chemoresistance. Therefore, the usage of inhibitors of this pathway provides an opportunity by dual targeting both cancer cells and cancer-associated stromal components [159]. The blockage of the PI3K pathway can occur at multiple sites, so there are several classes of inhibitors with application in the treatment of PC. The mTOR kinase inhibitors (e.g., everolimus) represent the classes used to decrease the progression of the disease to end stage and can also increase the efficacy of chemotherapy treatment with gemcitabine. It is better to use agents which can inhibit both mTORC1 and mTORC2 because they showed increased effectiveness [160].

The first option of PC treatment is surgical removal of the tumor followed by chemotherapy with gemcitabine. When surgery is not possible, but patients have a positive performance status, a combination of gemcitabine, FOLFIRINOX, and nanoparticle-bound (nab) paclitaxel is administered [161]. However, the prognosis remains poor, and chemotherapeutic drugs have proven to have only palliative roles in PC patients with unresectable or metastasized disease [162]. Nevertheless, hope arises from the molecular mechanisms involved in development and progression of pancreatic cancer and the new drugs that can interfere with crucial signaling pathways [163].

Alpelisib (BYL719, NVP-BYL719, Vijoice, Novartis Pharmaceuticals) is a drug administered orally that has proven to be very efficient when associated with fulvestrant in human epidermal growth factor receptor 2 (HER2)-positive breast cancer in advanced stages, efficiently inhibiting PI3Kα [164]. The SOLAR-1 phase III study led to the approval of this small molecule that can specifically inhibit p110α by the Food and Drug Administration (FDA) in 2022, recommending its use in PIK3CA-related overgrowth spectrum [164,165]. These promising results have encouraged the hypothesis that alpelisib can successfully treat PC as well, especially taking into consideration that the PI3Kα isoform has a major role in metastatic evolution [166]. An in vitro study performed on human and murine samples has shown that the inhibition of PI3Kα selectively reduced C36:2 PI-3,4,5-P3 and avoided macrophage accumulation. Thus, these results suggested that PI3Kα inhibition by alpelisib could provide new perspectives in averting the evolution towards macro-metastases [166]. An ongoing phase I clinical trial includes 15 participants diagnosed with pancreatic adenocarcinoma who were administered alpelisib in combination with gemcitabine and nab-paclitaxel [167]. The results of the study are yet to be published.

A phase Ib multicenter study aimed to investigate the effects of buparlisib (BKM120, NVP-BKM120, Selleck Chemicals), a pan-PI3K inhibitor in patients with solid tumors (triple-negative breast cancer, ovarian, cutaneous melanoma, colorectal cancer, non-small cells lung cancer), including in subjects with pancreatic cancer [168]. The study comprised 113 participants recruited from seven hospitals from the USA. The patients were administered buparlisib orally in combination with trametinib (an oral inhibitor of MEK1/2). During the trial, nine dose combinations were tested. The most common side effects were dermatological and gastrointestinal. Unfortunately, in pancreatic cancer patients, the activity of the drug was minimal. However, taking into consideration the promising results obtained in ovarian cancer patients, this inhibitor should be further investigated for PC as well [168].

A recent study analyzed the effects of this drug in association with MK-2206 (AKT inhibitor). The research was performed on 10 PDAC cell lines and showed that BKM120 promoted apoptosis or necrosis in all cell lines by inhibiting PI3K. On the other hand, buparlisib effect is not only dose-dependent but can also be influenced by AKT2 expression. The results of the study led by Ma X. [169] indicate that several factors must be taken into consideration when choosing a PI3K/AKT inhibitor, including target gene mutations.

NVP-BKM120 was also investigated in a previous study combination with mFOLFOX6 in a phase I, single center clinical trial that included 17 patients with advanced solid tumors and metastatic pancreatic cancer [170,171]. The aim of the study was to determine the tolerability of buparlisib in association with mFOLFOX6, and the results of the study showed that this combination presented elevated toxicity with neutropenia, fatigue, leukopenia, hyperglycemia, and thrombocytopenia as the most common side effects and established a maximum tolerated dose of 40 mg BKM120 per day compared to the well-tolerated 100 mg dose when administered alone or in other combinations [171].

A multicenter phase I study by Hong DS et al. analyzed PX-866 effects in patients with advanced solid tumors. Sonolisib or PX-866 (Cascadian Therapeutics), a synthetic PtdIns-3-kinase inhibitor, was administered to patients with colorectal cancer, melanoma, ovarian, lung cancer, and pancreatic neuroendocrine carcinoma among others. The aim of this study (the first clinical trial for PX-866) was to investigate its pharmacokinetics and to determine the maximum tolerated dose, as well as antitumor activity [172,173]. The results showed that the patients with incurable cancers included in this research tolerated a daily dose of 8 mg well. Moreover, the study that included 84 patients with advanced solid tumors revealed that PX-866 has promising potential, being associated with prolonged stable disease [173].

Copanlisib (BAY 80-6946, Aliqopa), a PI3K inhibitor, has been studied in a phase I clinical trial that included 57 patients with advanced cancers [174]. The results of the clinical study have shown that this inhibitor that acts preferentially on p110α and p110δ isoforms was well tolerated. In addition, at a dose of 0.4 mg/kg, intravenous administration of copanlisib is also safe for diabetes patients. Additionally, it displayed antitumor potential in advanced malignancies [175].

Pictilisib (GDC-0941, Genentech Inc.) has previously displayed great potential as an antitumor agent when studied on human tumor xenograft murine models, proving to be efficient in glioblastoma and ovarian cancer [176]. In the first-in-human phase I study, which was performed on 60 participants with advanced solid tumors (including pancreatic adenocarcinoma), this orally administered drug exhibited antitumor activity at doses higher than 100 mg per day. Furthermore, this clinical trial led to the recommended dose of 330 mg daily for a second phase study [177].

Another clinical trial revealed that GDC-0941 showed great antitumor potential in pancreatic cancer, determining tumor reduction [178]. On the other hand, the results of a phase I study showed that pictilisib (GDC-0941, RG7621), a pan-Pi3K inhibitor, had minimal antitumor efficiency and was not well tolerated in association with cobimetinib (GDC-0973, MEK inhibitor), despite showing great potential in pre-clinical studies [179]. The study included 178 participants (109 male and 69 female patients) and was terminated before the initiation of phase IIb because of the inability to find a recommended dose [179,180].

Perifosine (KRX041, NSC639966) is an allosteric AKT inhibitor through binding to the PH domain of AKT [181]. This alkylphospholipid has been studied in several clinical trials after promising results were obtained in research performed on animal models. Xin Y. et al. showed that perifosine inhibits S6K1–Gli1 signaling and prevents gemcitabine resistance in pancreatic cell cultures [182]. A phase II study that included 10 patients with metastatic PC was discontinued because of the severe adverse reactions along with no significant improvement in overall survival [183]. Another phase II clinical trial included 19 subjects with advanced PC who received 36 cycles of perifosine. After 6 months, 5 of the 17 eligible patients were alive, but the study was discontinued because the disease still had a high progression rate [184].

Uprosertib (GSK2141795) is an AKT inhibitor [185] which was investigated in a non-randomized clinical study that included patients with solid tumors, including PC patients in advanced stages, who were administered uprosertib in continuous/intermittent combination with trametinib [186]. The study was terminated early because the combination was not well tolerated, and no clinical improvements had been achieved [187].

Afuresertib (GSK2110183), a AKT1, 2, 3-competitive inhibitor [188,189], was studied in a phase I/II study in patients with solid tumors (including PC) and multiple myeloma in association with trametinib [190]. Phase I of the study aimed to assess the safety and maximum tolerated dose of both drugs, as well as their antitumor effects and pharmacokinetics, while phase II of the study included 11 participants with solid tumors, but the full results of the research are yet to be published [190,191].

In a study performed by Pan Y. et al., oleandrin (PBI-05204), a cardiac glycoside extracted from Nerium Oleander [192], displayed inhibitory effects on pAKT expression as well as on pS6 and p4EPB1 in human pancreatic PANC-1 cells [193]. Newman R.A. et al. had previously shown that this lipid-soluble compound determines cell death via autophagy [194]. Another study performed on Panc-1, BXPC3, and MiaPaca human pancreatic cell lines and a Panc-02 rodent cell line showed that Panc-1 responded the best to oleandrin administration, pancreatic cell sensitivity being dependent on the α3 subunits of Na-K ATPase [195]. A first-in-human phase I study evaluated the tumoral response of 46 patients with solid tumors, including two subjects diagnosed with pancreatic carcinoma, who had more than 4 months of stable disease. The study concluded that oleandrin had good tolerability and recommended a dose of 0.2255 mg/kg/day for an eventual phase II study [196]. A phase II single arm clinical trial that included 42 participants (38 were analyzed) with stage IV metastatic pancreatic cancer evaluating oleandrin safety and antitumor efficiency revealed that although the drug was well tolerated (gastrointestinal symptoms were most frequent), the progression-free survival was 56 days, and only 10 patients survived more than 4.5 months [197].

Sirolimus (rapamycin, AY-22989, RAPA, SILA, WY090217) is a macrolide that has been proven to successfully inhibit mTOR in preclinical models. In clinical trials that included patients with advanced cancers, including PC, sirolimus was combined with VEGFR kinases, such as sorafenib and sunitinib [198]. A phase II clinical trial included 47 patients with advanced pancreatic cancer, out of which 31 completed the study. Each patient was administered 5 mg of Sirolimus daily. The overall survival rate was 6 months [199]. A multi-institutional phase II clinical study included 33 patients with metastatic pancreatic ductal adenocarcinoma that did not respond to chemotherapy and who were administered a daily dose of 10 mg of everolimus (RAD001, Afinitor, Novartis) daily [200]. Everolimus is an oral inhibitor of the mTOR pathway that shows great promise in several types of cancers, in vitro and in vivo [201]. The patients included in this study tolerated the drug well, with hyperglycemia and thrombocytopenia having the most frequent negative reactions. However, the data obtained reflected that monotherapy with everolimus does not significantly impact the outcome of the disease, with the patients having a progression-free survival of 1.8 months, while the overall survival was 4.5 months. These results indicate that future therapeutic strategies should associate everolimus with drugs that target upstream components of the PI3K/AKT/mTOR signaling pathway [200].

A phase II study that included 31 patients with advanced pancreatic adenocarcinoma has shown that the combination of everolimus and capecitabine is safe, does not present toxicity, and provides an overall survival rate of 8.9 months. The results are promising, especially taking into consideration that everolimus cannot be associated with a full dose of the conventional chemotherapeutic drug administered in pancreatic cancer patients, gemcitabine [202].

Interestingly, metformin, a drug currently used to treat diabetes, could inhibit the mTOR pathway through AMPK activation [203]. However, a phase II study that included 121 participants with metastatic pancreatic tumors who randomly received gemcitabine, erlotinib (tyrosine kinase inhibitor-TKI), and metformin or gemcitabine, erlotinib, and placebo [204,205] aimed to assess the effects and the safety of concomitantly inhibiting both PI3K and MAPK signaling pathways at the same time as administering chemotherapy, starting from the hypothesis that the inhibition of only one signaling pathway is insufficient for significant results [205]. However, the results of the study indicated that metformin did not enhance the antitumor effect of gemcitabine and erlonitib [205]. On the other hand, an ongoing phase I/II study focused on metformin effects as a single agent or combined with sirolimus in patients with pancreatic cancer who previously received chemotherapy with FOLFIRINOX [206]. The study included 22 patients: 11 of them received only metformin, and the other 11 received the combined therapy. The trial revealed that metformin was well tolerated by the patients included in both arms of the study and improved the overall survival [207].

RX0201 or Archexin, an oligonucleotide that acts as an AKT antisense, is currently being studied in a phase II clinical trial [208,209,210]. A total of 250 mg/m^2^/day was administered to each patient with metastatic pancreatic cancer for 2 weeks followed by 1 week of resting. The drug produced by Rexahn Pharmaceutical was administered orally to 31 participants of the study, after prior use of gemcitabine. The results of the clinical trial are yet to be published [210].

MK-2206 (Merck & Co., Inc.) is a strong allosteric AKT inhibitor of the 1/2/3 isoforms [211] and has shown encouraging results in studies performed on human pancreatic cancer cell lines Mia PaCa-2 and Panc-1, not only inhibiting cell proliferation and promoting cancer cell apoptosis but also augmenting the cytotoxic effect of chemotherapy when administered in combination with gemcitabine [212]. Similar results were obtained in research conducted on Panc265 and Panc253 (patient-derived pancreatic cancer xenograft models), which demonstrated that MK-2206 combined with dinaciclib (a cyclin-dependent kinase inhibitor) inhibits tumor growth in pancreatic cancer [213]. Unfortunately, a phase I clinical trial did not show the same outcome as the preclinical studies [214].

Similar to MK-2206, triciribine phosphate monohydrate (NSC 154020, TCN) acts as a potent inhibitor of AKT 1/2/3 isoforms [215]. A study performed on MiaPaCa-2 cells indicated that this drug increases the cytotoxic effect of gemcitabine and promotes cell growth inhibition, acting synergically with chemotherapy [216].

Another study performed by Garrido-Laguna et al. aimed to test the efficiency of temsirolimus (WAY-CCI799), an inhibitor of the mTOR pathway, in pancreatic cancer using xenografts. The authors of the aforementioned research aimed to use preclinical models to investigate the potential of temsirolimus administered daily in a 5 mg dose. Their results showed that a positive response was obtained in 25% of the cases; however, these data did not translate in the clinical trial which was integrated with the preclinical investigation [199]. A clinical trial included five patients with advanced pancreatic cancer who received 25 mg of temsirolimus (CCl-779, Torisel) weekly and a second group of 16 patients that were administered 30 mg of everolimus weekly in combination with 150 mg of erlotinib daily. Unfortunately, neither inhibitor improved the outcome [217]. A phase I/II clinical trial investigated the effect of temsirolimus combined with gemcitabine in 30 patients with advanced/metastatic pancreatic cancer, concluding that while the side effects can be tolerated, the clinical effects are absent [218].

Another mTOR inhibitor, ridaforolimus (AP23573, MK-8669), was combined with bevacizumab and administered to 17 patients with advanced cancers, including two patients with pancreatic adenocarcinoma. The results of this phase I clinical trial were promising, with the drug combination prolonging stable disease, although caution is needed regarding patient selection (most severe adverse reaction was bowel perforation) [219].

Vistusertib (AZD2014) is an oral dual mTORC1/C2 inhibitor that has shown great potential in preclinical studies performed both in vitro and in vivo. In the first clinical trial that investigated the pharmacokinetics of this ATP competitive inhibitor, the recommended dose was determined to be of 50 mg twice a day, and it was revealed that a pancreatic cancer patient had a partial response [220]. Several clinical trials are still recruiting patients.

A phase I study included 32 patients with advanced/metastatic cancer who were administered a daily dose 100–500 mg of LY2780301, a p70S6K/AKT inhibitor. LY2780301 was found to have limited antitumor effects [221].

On the other hand, dual inhibitors of the PI3K/mTOR pathway, such as NVP-BEZ235 (Dactolisib, Selleck Chemicals), have proven to be more effective. A research study by Awasthi N. et al. studied its effects in pancreatic ductal adenocarcinoma, performed in vitro and in vivo on murine xenografts, and showed that the drug displayed antitumor activity in both situations. Moreover, when administered together with other drugs such as gemcitabine and endothelial monocyte activating polypeptide II, NVP-BEZ235 expressed even greater potential [222]. However, in a study realized on pancreatic ductal adenocarcinoma cell lines, BEZ235 also upregulated the extracellular signal-regulated kinase (ERK) pathway, determining its overactivation [223]. The ERK pathway plays a significant role in tumorigenesis, being involved in invasion and tumor metastasis [224]. Furthermore, it has been demonstrated that the efficiency of PI3K/mTOR inhibition by BEZ235 directly correlates with ERK overactivation, an aspect which must be taken into consideration regarding its clinical use [223].

Previous studies show that cancer stem cells have an important role in patient relapse, with PI3K/AKT/mTOR as well as the Sonic Hedgehog signaling pathway being reactivated in the process. Sharma N. et al. have demonstrated that and association of NVP-BEZ235 and NVP-LDE225 could offer new hopes in pancreatic cancer treatment [225]. NVP-LDE225 (Sonidegib, Novartis) is an oral drug that acts as an antagonist for smoothened receptor, thus inhibiting the Hedgehog pathway [226,227,228].

The antitumor activity of Voxtalisib (SAR245409, XL765), a potent dual inhibitor of PI3K and mTORC1/C2 [229,230], was investigated in a phase I clinical trial that included 146 patients with solid tumor (pancreatic, colorectal, ovarian, etc.). Voxtalisb was administered orally in association with primasertib (MEK inhibitor), the results of the study revealing that this combination was poorly tolerated and had narrow antitumor effects [230]. On the other hand, a phase I trial conducted by Papadopoulos K.P. revealed that voxtalisib has an attainable safety profile, and 48% of the 83 patients with solid tumor included in the study had stable disease after XL765 treatment [231].

A four-arm clinical trial investigated the antitumor activity of gedatolisib (PF05212384, PKI-587) and PF-04691502, two dual PI3K/mTOR inhibitors that were combined with Irinotecan or PD-0325901 (MEK inhibitor) and administered to patients with advanced solid tumors. No significant improvements were observed in pancreatic cancer patients [232]. The first clinical trial investigating the safety of SF1126, another dual PI3K/mTOR inhibitor, revealed that this peptidic drug is well tolerated by patients with advanced solid tumors and shows great potential as a target in treatment of malignancies. However, further studies are needed [233].

RAD001 in advanced neuroendocrine tumors (RADIANT-1) was performed in 11 countries and 36 centers and was a phase II clinical trial that included 160 patients with metastatic pancreatic neuroendocrine tumors who showed no improvement after chemotherapy focused on the potential of everolimus (RAD001). A total of 115 patients were orally administered 10 mg of Everolimus daily, while 45 patients were administered 10 mg of RAD001 together with octreotide LAR (long-acting release). The results of the clinical trial showed that everolimus presented antitumor activity in pancreatic neuroendocrine tumors. Moreover, the obtained data suggested that the combination therapy is more efficient compared to everolimus administration alone, since octreotide LAR can decrease IGF-1 levels in patients with malignant tumors, thus reducing everolimus resistance (IGF-1 upregulation could lead to it) [201].

RADIANT-2 and RADIANT-3 phase III multicenter trials confirmed the results [234]. RADIANT-2 was a double-blind, randomized study that included patients with low- or intermediate grade neuroendocrine pancreatic tumors who were divided into two groups: The first group received 10 mg of everolimus daily combined with 30 mg of octreotide LAR per month, while the second group was administered a placebo together with octreotide LAR. The results showed that patients who received everolimus had a better progression-free survival compared to the second group [235]. The potential of RAD001 was confirmed by RADIANT-3, a randomized, double-blind study that included 410 patients with pancreatic neuroendocrine tumors from 18 countries and 82 centers. This placebo-controlled clinical trial demonstrated that everolimus significantly increased progression-free survival versus the placebo [236]. These promising results lead to the approval of everolimus for progressive (unresectable or metastasized) pancreatic neuroendocrine tumors treatment in 2011 [234].

Omipalisib (GSK458, GSk212458) is an ATP-competitive dual PI3K/AKT/mTOR inhibitor proven to be effective in association with trametinib when tested on pancreatic cell lines, demonstrating antitumor activity [237]. In the first-in-human clinical trial 170 patients (two of which were diagnosed with pancreatic cancer) were enrolled. The aim of the phase I study was to establish the maximum tolerated dose for the drug. Diarrhea, skin rash, and fatigue were the most common side effects of GSK458. The results of the study showed that patients tolerate 2.5 mg/day of omipalisib [238].

The presented data suggest that further investigating the complex molecular mechanisms and intricated signaling pathways could offer new therapeutic perspectives for clinicians treating pancreatic cancer patients. In this review, we focus on PI3K/AKT/mTOR inhibitors that have shown great anti-tumor potential in studies performed on xenograft models and pancreatic cell lines and inhibitors tested in clinical trials. Although numerous inhibitors of this key signaling pathway have been developed, a small number have been shown to be efficient in pancreatic cancer in preclinical studies, and even fewer were administered in clinical research. Many of them are not yet allowed to be used on patients because of their toxicity and inhibition of other crucial signaling pathways. Therefore, solutions must be found in order to test the real impact of these drugs in a clinical setting [127] (Table 1).

## 6. Conclusions

Pancreatic tumors arise from the endocrine and exocrine areas and may in the future become one of the leading causes of deaths worldwide. *KRAS*, *CDKN24*, *TP53*, and *SMAD4* are the most common gene mutations detected in PC patients. PDAC, the most frequent type of PC, has KRAS mutations in 95% of cases, which are correlated with PC progression. Certain eating habits, such as animal protein consumption, sedentary lifestyle, smoking, and alcohol consumption are involved in the PC pathogenesis. The link between PC and risk factors seems to be chronic inflammation, which mediates PC-TME development and further EMT transition, leading to tumor growth, angiogenesis, metastasis, and even chemoradiotherapy resistance. PI3K/AKT/mTOR is one of the most mutated signaling pathways in human malignancies including PC, being activated by various factors such as cytokines, hormones, and growth factors. Therefore, inhibitors of this signaling pathway have been discovered, and some of them have already been used in clinical trials, with promising results in terms of survival rate. Everolimus is an mTOR inhibitor is used in clinical studies for metastatic and advanced PDAC patients. The results seem to be promising only in advanced PDAC patients, providing an overall survival rate of 8.9 months. Clinical trials using everolimus in advanced neuroendocrine tumors reported that the drug displayed antitumoral activity. Moreover, promising results come from cell lines and animal studies, where PI3K/AKT/mTOR inhibitors associated with ERK/ MAPK or Sonic Hedgehog inhibitors seem to be more effective. In conclusion, investigating this complex signaling pathway could offer new therapeutic perspectives for clinicians treating PC patients.

## Figures and Tables

**Table 1:** PI3K/AKT/mTOR inhibitors used in pancreatic cancer studies.

Inhibitor	Target	Mechanism	Study	References
Alpelisib (BY719, NVP-BYL719)	PI3K	ATP competitive	Solar-1 phase III	[164,165]
Buparlisib (BKM120, NVP-BKM120)	PI3K	ATP competitive	Phase Ib multicenter	[168]
Sonolisib (PX-866)	PI3K	Allosteric	Multicenter phase I	[172,173]
Copanlisib (BAY80-6946)	PI3K	ATP competitive	Phase I clinical trial	[174,175]
Pictilisib (GDC-0941, RG7621)	PI3K	ATP competitive	Xenograft murine models	[176]
Pictilisib (GDC-0941, RG7621)	PI3K	ATP competitive	First-in-human phase I	[177,178,179]
Perifosine (KRX041, NSC639966)	AKT	Allosteric	Cell lines	[182]
Perifosine (KRX041, NSC639966)	AKT	Allosteric	Phase II, clinical trial	[183,184]
Uprosertib (GSK2141795)	AKT	ATP competitive	Non-randomized clinical study	[185,186]
Afuresertib (GSK2110183)	AKT	ATP competitive	Phase I/II	[190,191]
Oleandrin (PBI-05204)	AKT	Information not available	Pancreatic cells	[193,194,195]
Oleandrin (PBI-05204)	AKT	Information not available	First-in-human phase I/II	[196,197]
MK-2206	AKT	Allosteric	Cell lines	[169,215,216]
MK-2206	AKT	Allosteric	Xenograft models	[212,213]
MK-2206	AKT	Allosteric	Phase I clinical trial	[214]
Archexin (RX0201)	AKT	Antisense oligonucleotide	Phase II clinical trial	[210]
LY2780301	AKT	ATP competitive	Phase I	[221]
Sirolimus (AY-22989, RAPA, SILA, WY090217)	mTOR	Allosteric	Phase II clinical trial/ multi-institutional phase II	[199,200]
Everolimus (RAD001)	mTOR	Allosteric	Phase II/clinical trial	[201,202,233,235,236]
Metformin	mTOR	Information not available	Phase I/II	[204,205,206,207]
Temsirolimus (CCI 779, WAY-CCI779)	mTOR	Allosteric	xenografts	[199]
Temsirolimus (CCI 779, WAY-CCI779)	mTOR	Allosteric	Clinical trial/phase I/II	[217,218]
Ridaforolimus (AP23573, MK-8669)	mTOR	Allosteric	Phase I clinical trial	[219]
Vistusertib (AZD2014)	mTOR	ATP competitive	Clinical trial	[220]
Dactolisib (NVP-BEZ235, BEZ235)	PI3K/mTOR	ATP competitive	Cell lines	[222,225]
Voxtalisib (SAR245409, XL765)	PI3K/mTOR	ATP competitive	Phase I clinical trial	[230,231]
Gedatolisib (PF05212384, PKI-587)	PI3K/mTOR	ATP competitive	Four-arm-clinical trial	[232]
PF-04691502	PI3K/mTOR	ATP competitive	Four-arm-clinical trial	[232]
SF1126	PI3K/mTOR	ATP competitive	Clinical trial	[233]
Omipalisib (GSK458, GSK2126458)	PI3K/mTOR	ATP competitive	Clinical trial	[238]
